# Comparative Assessment of Environmental DNA and Bulk-Sample Metabarcoding in Biosecurity Surveillance for Detecting Biting Midges (Ceratopogonidae)

**DOI:** 10.3390/insects16060564

**Published:** 2025-05-27

**Authors:** Jieyun Wu, Dongmei Li, Rebijith K. Balan, Sherly George, Lora Peacock, Chandan Pal

**Affiliations:** 1 Plant Health and Environment Laboratory, Biosecurity New Zealand, Ministry for Primary Industries, P.O. Box 2095, Auckland 1140, New Zealand; jieyun.wu@auckland.ac.nz (J.W.); dongmei.li@mpi.govt.nz (D.L.); rebijith.balan@mpi.govt.nz (R.K.B.); sherly.george@mpi.govt.nz (S.G.); 2 School of Biological Sciences, The University of Auckland, 23 Symonds Street, Auckland 1010, New Zealand; 3 Surveillance and Incursion Investigation (Plants and Environment), Ministry for Primary Industries, P.O. Box 2526, Wellington 6140, New Zealand; lora.peacock@mpi.govt.nz; 4 Zespri International Limited, Mount Maunganui 3116, New Zealand

**Keywords:** *Culicoides*, insect vector, environmental DNA, early detection, high-throughput sequencing, metabarcoding, pest surveillance, biosecurity

## Abstract

Biting midges, *Culicoides* spp. (Diptera: Ceratopogonidae), are significant vectors capable of transmitting arboviruses, such as bluetongue virus, to livestock. New Zealand is free of *Culicoides*, and a national surveillance programme is in place for the early detection of an incursion. Traditionally, insect trap samples from the surveillance programme are analyzed using morphology-based diagnostics under microscopes, which is time-consuming and relies on specialized taxonomic expertise. Here, we assessed the effectiveness of DNA metabarcoding using insect bulk samples and environmental DNA (eDNA) from liquid samples collected in surveillance traps. Two Cytochrome oxidase I (COI) barcoding primer sets were employed to study biodiversity and detect exotic species. The results indicated that DNA metabarcoding with homogenized insect bulk samples had a higher overall detection accuracy rate (over 81% for both primer pairs) compared to ethanol fluid-derived eDNA samples from traps (68.42% and 55.26% for the primer sets LCO1490/HCO2198 and mlCOIintF/jgHCO2198, respectively) based on congruence with morphological identification. Detection failures were likely due to eDNA extraction issues or low target species abundance. Both approaches showed similar insect community composition and diversity in the surveillance trap samples, suggesting the potential of DNA metabarcoding for biosecurity surveillance and biodiversity assessments. Overall, DNA metabarcoding using bulk insect samples could enhance the efficiency of *Culicoides* surveillance, reducing workload and screening time.

## Simple Summary

*Culicoides* biting midges are vectors that can transmit pathogens, causing many diseases of veterinary concern, including bluetongue virus, which significantly affects primary production animals. New Zealand is free of these midges, and national surveillance programmes are in place to detect them and their vector-borne diseases early. Traditionally, insects from these trap samples are identified under the microscope, which is slow and requires specialized taxonomic expertise. This study compared two molecular approaches, DNA metabarcoding using insect bulk samples from traps and environmental DNA (eDNA) from trap liquids, considering morphological outcomes for detecting biting midges and *Culicoides* identification. The DNA metabarcoding of bulk insect samples was more accurate (over 81%) compared to eDNA samples from trap fluids (55.26–68.42%) in matching morphological outcomes. Both methods provided similar information about insect communities in traps, suggesting non-destructive eDNA metabarcoding could be useful for biosecurity surveillance and biodiversity monitoring. Overall, DNA metabarcoding with insect bulk samples could make the diagnostic process and surveillance strategy more efficient, reducing workload and screening time.

## 1. Introduction

*Culicoides* spp. (Diptera: Ceratopogonidae) are biting midges that are the world’s smallest blood-feeding flies, measuring only 1–3 mm in size [1]. Many of their distinguishing morphological features can only be examined under a microscope. Currently, over 1400 *Culicoides* species are recognized within the genus, and they occur globally with the exception of New Zealand, Patagonia, Hawaii and Antarctica [2]. They can vector more than 50 arboviruses, including some important and severe pathogens affecting livestock and horses, such as bluetongue virus, African horse sickness virus, Akabane virus, and bovine ephemeral fever virus [2,3,4].

Although most flight ranges of *Culicoides* are very short distances, they are capable of being dispersed passively over much longer distances by wind [5,6,7]. Windborne dispersal has linked to outbreaks of bluetongue virus in the Mediterranean, North Africa and Northern Europe; African horse sickness in the Middle East; and epizootic haemorrhagic disease in Israel [7,8,9,10,11,12]. It is also believed that the introduction of novel serotype of bluetongue virus into Australia occurred via windborne dispersal from regional neighbours, such as Indonesia or other islands of Timor, across the Timor Sea [13,14].

New Zealand’s current freedom from *Culicoides* species and subsequent arboviruses has been possible due to its geographical isolation and robust biosecurity system. Maintaining a *Culicoides*-free status minimizes the risk of introducing these diseases and protects the health and productivity of New Zealand’s livestock. However, substantial evidence suggests that many insect species (e.g., moths and butterflies), fungal spores, pollen, and seeds were introduced in New Zealand via windborne dispersal [15,16,17,18]. Thus, the risk of a windblown *Culicoides* incursion from Australia and other neighbouring countries is considered possible, which has resulted in the implementation of a national surveillance programme for both the vector and its arboviruses [19].

Over the years, traditional pest surveillance tools have been improved. For example, light traps combined with green LEDs, octenol, and carbon dioxide attractants have been effective in attracting various midge species [20]. However, the small size of the midges trapped and other by-catches leads to a time-consuming and labour-intensive screening process under the microscope, and the taxonomic expertise and resources required to identify each individual has been a significant challenge in the current surveillance programme [21,22]. On average, in a typical year, 15,000–20,000 insects are collected from the light traps.

To mitigate this challenge, molecular approaches, such as DNA metabarcoding in combination with high-throughput sequencing, can offer a more efficient, cost-effective, reliable alternative option to identify the insect species from the insect trap samples. This could be achieved using mixed insect bulk samples, as well as liquid medium from the trap containing the shed environmental DNA (eDNA) [23,24,25,26,27]. Recently, an eDNA metabarcoding approach showed inconsistency in identifying *Culicoides* specimens at the species level from the light traps filled with saturated salt solution [28]. It is also noteworthy that the high sensitivity of DNA metabarcoding makes it able to detect rare species in environmental samples to reduce false negatives [29]. This approach has been widely implemented for the detection of a variety of invasive species of flying insects, such as mosquito, stink bugs, etc., from various insect traps, leaf surfaces, or water samples [30,31,32].

The main objective of this study was to explore the insect biodiversity in the trap samples and detection of Ceratopogonidae species while assessing the effectiveness of performing DNA metabarcoding using homogenized insect bulk samples and environmental DNA (eDNA) metabarcoding using liquid samples collected in insect traps in a *Culicoides* surveillance programme in New Zealand in 2020.

## 2. Materials and Methods

### 2.1. Study Sites and Sample Collection

**Figure 1:**
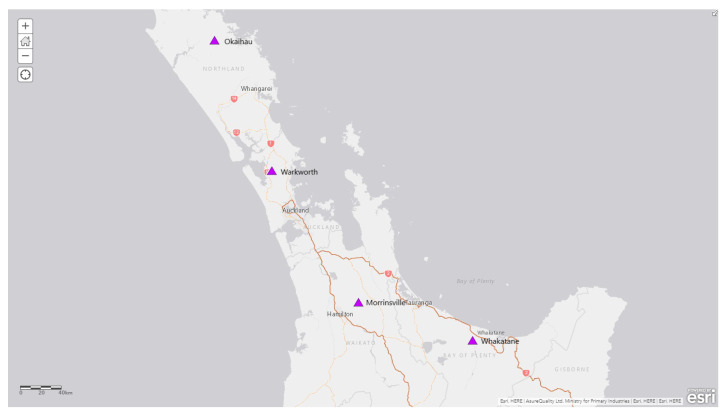
An overview of all sampling locations of the study sites across New Zealand. See Table 1 for the detailed metadata for the samples collected from those sites.

A total of four cattle farms with predominantly high cattle numbers from four districts (i.e., Morrinsville, Okaihau, Warkworth, and Whakatane) from the 2020 surveillance programme were selected for this study (Figure 1). The surveillance traps were deployed at those sites from the start of February until the beginning of April. The green LED light traps incorporated with CO_2_ (CO_2_ gas was in cylinders, and 400 mL of CO_2_ gas per min was released by the light trap) and octenol attractant were deployed on three consecutive nights over nine or ten weeks (Okaihau and Whakatane for nine weeks, and Morrinsville and Warkworth for ten weeks), and insect traps samples were collected every week.

The preservative fluid used in the trap was ethanol-based (70% alcohol), and all the trap contents, with their by-catches, were transported to the laboratory for morphological identification and eDNA extraction. The insect trap liquid was filtered into a sterilized sampling bottle by a sterilized 0.2 µm filter. The insect specimens derived from the sample were inspected using morphological characteristics under the microscope [33,34]. For each trap, the total number of insects and the prevalence of *Culicoides* species were recorded.

As *Culicoides* species are absent in New Zealand, the prevalences of New Zealand’s native species of Ceratopogonidae, which belong to the same family as exotic *Culicoides* species, were used as a proxy to examine the detection accuracy of DNA metabarcoding approaches.

Both the samples of ethanol fluid from traps and insect body bulk after inspection were stored at 4 °C until further processing. Overall, a total of 38 insect trap samples (2 sites × 10 weeks + 2 sites × 9 weeks = 38 samples) were collected for two sample types.

### 2.2. eDNA Capture and Extraction

To avoid DNA degradation and increase the detection probability [35], within 48 h after sample inspection, insect trap liquid samples were filtered through 0.2 μm sterile nitrocellulose filters (Ahlstrom-Munksjo, Helsinki, Finland) with a vacuum pump using a filter holder (Rocker Scientific, New Taipei City, Taiwan). To prevent cross-contamination, after each sample, the filter holder was cleaned to destroy the residual DNA by soaking for 30 min in a 10% bleach solution and then rinsing with distilled water three times to prevent the build-up of bleach salts. The nitrocellulose filters were replaced with new ones after filtering each sample. The samples were then extracted and purified using the DNeasy Blood & Tissue kit (Qiagen, London, UK) following the manufacturer’s protocol. DNA was finally eluted in 100 μL of AE buffer. Prior to the filtration of the field samples, a negative control (sterile water) was used to examine for possible cross-contamination.

Additionally, each bulk sample of insect bodies was placed on one bacterial culture plate to air-dry the rest of the ethanol. Then, the bulk sample was placed into a grinding extraction bag and 10 mL of CTAB buffer (containing antifoam) was added into the bag. After thoroughly grinding the insects using the Homex grinder (Bioreba, Reinach, Switzerland), 1 mL of the ground mixture and 50 μL of Proteinase K were pipetted into 3 × 1.5 mL tubes (triplicates for each sample). Those samples were then incubated at 65 °C for 20–30 min in a thermomixer. Finally, after centrifugation at ≥10,000 RPM for 2 min to pellet the debris, eDNA was extracted from all samples using the Kingfisher mL workstation (Thermo Fisher, Waltham, MA, USA) with an InviMag Plant Kit (Invitek GmbH, Berlin, Germany) following the manufacturer’s protocol.

The quality of the extracted DNA for both insect trap fluids and insect bulk samples was assessed using a NanoDrop^TM^ spectrophotometer (Thermo Fisher Scientific, Waltham, MA, USA) and quantified using the Quant-iT Picogreen dsDNA assay kit (Thermo Fisher Scientific, Waltham, MA, USA).

### 2.3. PCR and Metabarcoding Sequencing

For both DNA sample types, DNA amplification within the COI subunit region was performed with two sets of primer pairs, LCO1490/HCO2198 [36] and mlCOIintF/jgHCO2198 [37], which were both modified to include the Illumina adapter sequences required for downstream sequencing processes based on the standard Illumina protocol [38]. PCR reactions for both primer pairs contained 5.4 μL of sterile water, 10 μL of Platinum^TM^ SuperFi II PCR Master Mix (Thermofisher, Waltham, MA, USA), 1 μL each of the forward and reverse primers (5 μM concentration), 0.6 μL of 50 mM MgCl_2_, 1 μL of BSA and 1 μL of DNA template. Reactions for the primer pair LCO1490/HCO2198 were held at 94 °C for 5 min, before 40 cycles at 94 °C for 15 s, 50 °C for 40 s, and 72 °C for 45 s, and finally extension at 72 °C for 7 min. The amplification protocol for the primer pair mlCOIintF/jgHCO2198 was as follows: 95 °C for 3 min, 40 cycles of 95 °C for 30 s, 50 °C for 30 s, and 72 °C for 1 min, and then final extension at 72 °C for 10 min.

After the PCR run, all amplified PCR products were checked for DNA integrity on 1.5% agarose gels in TAE buffer stained with SYBR safe (Life Technologies, Sunnyvale, CA, USA) and visualized using a Gel Doc Go Imaging system (BioRad, Hercules, CA, USA). All negative controls for both DNA extraction and PCR amplification were negative, indicating that no cross-contamination occurred. Triplicate DNA samples from each insect bulk sample were pooled for the following processes to reduce the number of sequencing samples and to ensure that insect trap liquid samples and insect bulk samples were comparable in terms of the sampling size.

After PCR amplification, each PCR product was individually purified using AMPure XP reagents (Beckman Coulter, Indianapolis, IN, USA), according to the manufacturer’s instructions. The DNA concentration of those amplified PCR products was also quantified using a Qubit^®^ dsDNA HS Assay Kit (Life Technologies, Sunnyvale, CA, USA). During the library preparation stage, a combination of Nextera XT A and B barcode dual indices (Illumina Inc., San Diego, CA, USA) was attached to the DNA from each sample. This approach ensured that the DNA from each sample could be identified by its unique DNA barcode. Amplicons from all the samples were composited together in equimolar concentrations and sequenced on an Illumina MiSeq instrument with 2 × 300 bp paired-end sequencing chemistry at the Auckland Genomics Facility, New Zealand. Raw sequencing data were deposited in the NCBI SRA (Sequence Read Archive) database with the BioProject ID PRJNA1249277.

### 2.4. In-House Reference Database of Ceratopogonidae Species

Over the past 6 years, a total of 90 COI sequences of 6 different genera (i.e., Leptoconops, Atrichopogon, Austrohelea, Dasyhelea, Forcipomyia, and Paradasyhelea) belonging to the Ceratopogonidae family were sequenced in-house. Using these sequences, an in-house COI barcoding database was generated, containing all known Ceratopogonidae species from New Zealand. This customized reference database was used for DNA-based taxonomic identification of Ceratopogonidae sequences.

### 2.5. OTU Analysis and Taxonomic Assignment

Bioinformatics analysis was conducted to process demultiplexed raw HTS sequences using the USEARCH tool [39]. In brief, paired-end reads were merged and filtered for quality using default parameters. Because the fragment of the PCR product of the primer pair LCO1490/HCO2198 was about 650 bp, the 2 × 300 bp paired-end sequencing could not recover the whole length of the fragment. Thus, only the forward reads for the LCO1490/HCO2198 primer pair were used, whereas only reads that merged both the forward and reverse reads and those that had a minimal length of 300 bp for the primer pair mlCOIintF/jgHCO2198 were used for further analysis.

After the removal of the replicate (i.e., dereplication) and singleton sequences, chimeric sequences were removed and DNA sequence reads were de novo clustered into operational taxonomic units (OTUs) at 97% similarity using the UPARSE algorithm. Reads were then mapped to the final list of OTUs to assign abundances to each OTU and generate the OTU table for each primer pair.

Taxa were assigned to each OTU using the USEARCH global alignment algorithm by aligning the OTU sequences against the in-house customized COI reference database of Ceratopogonidae. All hits above threshold for a minimum sequence identity of 95% and a minimum alignment length of 200 bp were collected. Meanwhile, if no reference sequence with identity > 95% was available for the OTU sequence, those sequences were queried again against the NCBI nr database (downloaded in August 2019) using the Basic Local Alignment Search Tool (i.e., BLAST+ 2.8.1) algorithm, with the following parameters: a maximum e-value of 0.05 and a minimum sequence identity of 95%. In both USEARCH and BLAST approaches, a match with the highest sequence identity and the longest alignment length was selected as the best hit and the corresponding taxa were assigned to that OTU.

### 2.6. Data Analysis and Visualization

To evaluate the accuracy and detectability of the metabarcoding-based approach, the prevalence of Ceratopogonidae species identified via the molecular approach was compared with the results obtained from traditional morphological screening. Detection accuracy for each combination of sample type (insect trap fluid versus insect bulk) and primer pair (LCO1490/HCO2198 versus MlCOIintF/jgHCO2198) was quantified and visualized using pie charts.

Then, to further determine the relationship between the detectability and performance of the eDNA approach and the relative abundance of Ceratopogonidae species within the total insect bulk, balloon plots were used to visualize the data of the detectability of the eDNA approach and ratio of the number of Ceratopogonidae species versus total number of insects identified at each sampling site across ten weeks. The performances of two COI universal primer pairs were visualized in pie charts using the number of OTU reads belonging to different common insect types, orders, and families. Finally, to visualize and assess variations in the composition of insect communities derived from insect trap liquid and insect bulk samples amplified by two COI primer pairs, HTS data were plotted using nMDS (non-metric multidimensional scaling) analysis of the Bray–Curtis dissimilarity matrix derived from those data. All of the abovementioned figures were generated using the ‘ggplot2’ and ‘vegan’ packages in R (version 3.6.0) [40,41].

## 3. Results

### 3.1. Morphological Identification of Insect Trap Samples

Under the microscope, a total 45,745 insects were found in 38 surveillance trap samples. No exotic *Culicoides* species were identified in any of the trap samples. However, native Ceratopogonidae species were found in 58% of the trap samples (22 out of 38 traps) (Table 1). Only 0.25% of trap catches (*n* = 114) were identified as belonging to the Ceratopogonidae family, with 83% of those individuals detected in the Whakatane district in the Bay of Plenty, where Ceratopogonidae species were found in all nine surveillance trap samples. All individual insects belonging to the Ceratopogonidae family were sent to an external taxonomic expert for further identification at the genus and species levels.

### 3.2. Detection Accuracy of eDNA Metabarcoding Approach

In practical applications, no single approach can accurately or consistently identify true positives or true negatives of the target species from mixed environmental samples. Morphological identification, DNA metabarcoding of bulk samples, and eDNA metabarcoding complement each other can be used in detecting and identifying target species in mixed environment samples [42,43]. However, in this study, detection accuracy was defined as how well the molecular methods detected and identified the target species found via traditional morphological methods, with congruence to morphological outcomes considered true positives or negatives.

**Figure 2:**
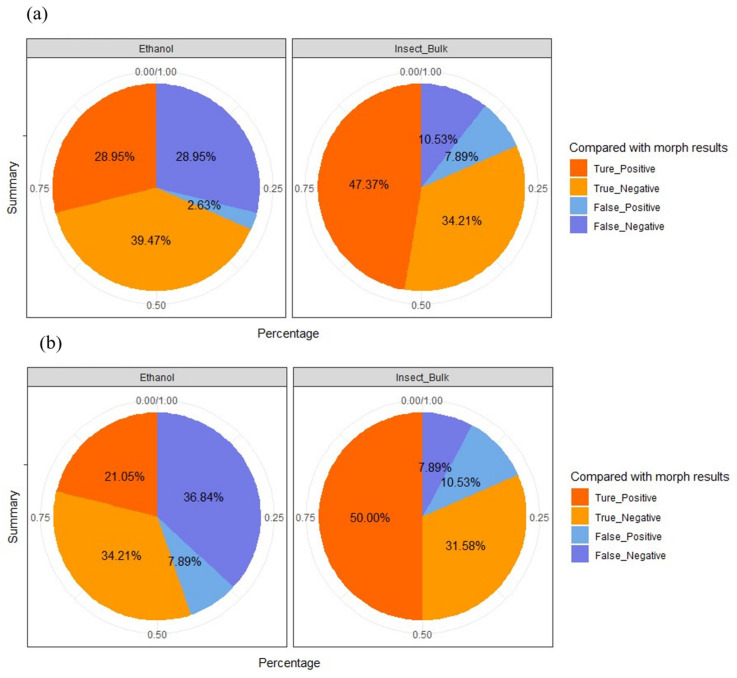
Pie charts showing the overall detection accuracy rate of the eDNA approach to determine the presence of Ceratopogonidae species at each study site based on different sample types (i.e., insect trap liquid or insect body bulk derived from the same trap sample) using (**a**) the primer pair LCO1490/HCO2198 and (**b**) the primer pair mlCOIintF/jgHCO2198, compared with the morphological identification.

DNA extracted from insect trap samples was run on the Illumina MiSeq high-throughput DNA sequencing platform. In total, 4.6 million reads by primer pair LCO1490/HCO2198 and 6 million reads by the primer pair mlCOIintF/jgHCO2198 that passed the quality filtering could be assigned to a sample. In that dataset, 557 and 6078 distinct unique operational taxonomic units (OTUs) with 97% DNA sequence similarity were identified for the primers LCO1490/HCO2198 and mlCOIintF/jgHCO2198, respectively. Among these, 486 and 1247 OTUs, respectively, could be assigned to taxonomic groups at the family level with 95% similarity to the COI reference sequences derived from the in-house database and the public databases. Taxonomic assignments of OTUs to Ceratopogonidae species were compared to morphological identifications to determine the detection accuracy rate of the molecular approaches via two different primers. Based on OTU counts from DNA metabarcoding sequences of trap ethanol fluids and homogenized insect bulk samples, the detection accuracy rates (i.e., true positive rates + true negative rates) for the primer pair LCO1490/HCO2198 (Figure 2a) were 68.42%, (*n* = 11 + 15) and 81.94% (*n* = 18 + 13), respectively.

In contrast, the overall detection accuracy rates using the primer pair mlCOIintF/jgHCO2198 (Figure 2b) were 55.26%, (*n* = 8 + 13) and 81.58%, (*n* = 19 + 12), respectively, for trap ethanol fluids and insect bulk samples. The results indicated that regardless of the primer sets used for amplification, in general, DNA metabarcoding approach using insect bulk correctly identified the presence of native Ceratopogonidae species in over 81% of insect traps samples (*n* = 31 samples out of 38) compared to the eDNA metabarcoding approach using insect trap ethanol fluid samples (overall accuracy rates of 68.42% and 55.26% using two separate primers). Overall, these findings suggest that the DNA-metabarcoding approach using insect bulk could detect the presence or absence of target species (Ceratopogonidae species in this case) from pest surveillance trap samples in the majority of the cases, while the detectability and accuracy of such an approach via eDNA metabarcoding using the liquid samples from insect trap requires further improvement.

### 3.3. Effect of Target Species Abundance on eDNA Detection

Due to the variation in the detection accuracy rate of the DNA metabarcoding approach observed from different sample types (trap ethanol fluid versus insect bulk) and different primer sets used for PCR amplifications, it was further determined whether the relative abundance of target species (Ceratopogonidae species as the indicator in this case) in the total insect communities obtained from the trap collection would impact the eDNA detection accuracy.

**Figure 3:**
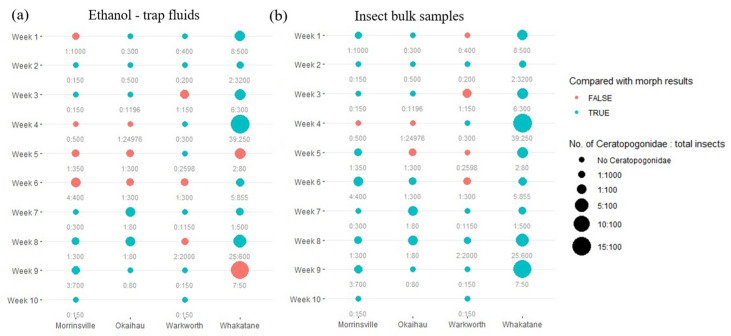
The relationship between the detection accuracy of the DNA metabarcoding approach derived from (**a**) trap ethanol fluids and (**b**) insect bulk samples using the primer pair LCO1490/HCO2198 and the relative abundance of Ceratopogonidae species present in each sample across the study site. Different columns represent data collected from different sampling sites; different rows represent data from different collection dates. The sites with no data are shown blank. The consistency between the traditional morphological results and molecular approach findings regarding the presence of Ceratopogonidae species is represented by the colour of the circle (blue represents a consistent observation; red represents an inconsistent observation). Additionally, the radius of each circle represents the amount of variation in the ratio of the number of Ceratopogonidae species versus the total number of insects found in each sample. The actual numbers of Ceratopogonidae species and total number of insects observed from each sample are labelled below each circle.

For the primer pair LCO1490/HCO2198, the detection (presence/absence) of Ceratopogonidae species failed via eDNA metabarcoding using trap fluid samples compared to morphological findings in 12 out of 38 trap samples (31.6%) (Figure 3a). In contrast, DNA metabarcoding using insect bulk samples had fewer detection failure cases (7 out of 38 trap samples; 18.4%) (Figure 3b). In insect bulk samples, false negatives (i.e., detected under the microscope but not via metabarcoding) and false positives (i.e., detected via metabarcoding but not under the microscope) contributed almost equally to the detection failures, unlike trap liquids, where detection failures occurred mainly due to false negatives. However, a trend of fewer detection failures was observed with an increase in the relative abundance of target species within the community. Notably, although classified as false positives when compared to trap catches under the microscopes, metabarcoding approaches detected the presence of Ceratopogonidae species in one liquid trap sample and three insect bulk samples that morphological approaches failed to detect.

**Figure 4:**
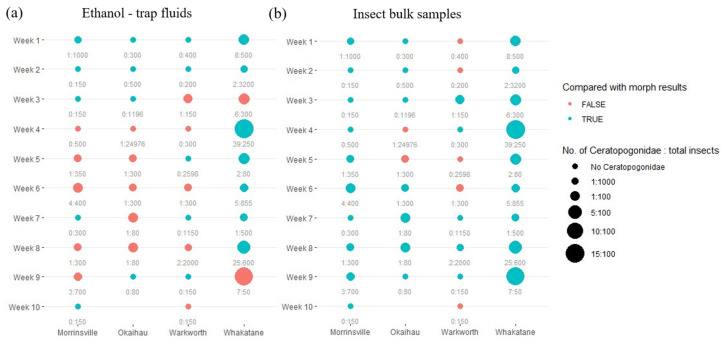
The relationship between the detection accuracy of the DNA metabarcoding approach derived from (**a**) trap ethanol fluids and (**b**) insect bulk samples using the primer pair mlCOIintF/jgHCO2198 and the relative abundance of Ceratopogonidae species present in each sample across the study site. Different columns represent data collected from different sampling sites; different rows represent data from different collection dates. The sites with no data are shown blank. The consistency between the traditional morphological results and molecular approach findings regarding the presence of Ceratopogonidae species is represented by the colour of the circle (blue represents an consistent observation; red represents an inconsistent observation). Additionally, the radius of each circle represents the amount of variation in the ratio of the number of *Ceratopogonidae* species versus the total number of insects found in each sample. The actual numbers of *Ceratopogonidae* species and the total number of insect observed from each sample are labelled below each circle.

In addition, regarding the observations made from the trap ethanol liquids (Figure 4a), the primer pair mlCOIintF/jgHCO2198 performed worse than those observed from the primer pair LCO1490/HCO2198 (Figure 3a). Similar to the previous observation, the eDNA metabarcoding approach using insect trap fluids was unable to replicate the outcomes achieved under the microscope and with the DNA metabarcoding approach using insect bulk samples (Figure 4a). However, the choice of different primers did not affect the detection accuracy of DNA metabarcoding on the insect bulk samples (Figure 3b and Figure 4b), as a similarly accurate observation was made for mlCOIintF/jgHCO2198 in comparison with the one derived from LCO1490/HCO2198.

### 3.4. Impacts of Primer Choices and Sample Types on the Recovery of Overall Taxonomic Diversity

**Figure 5:**
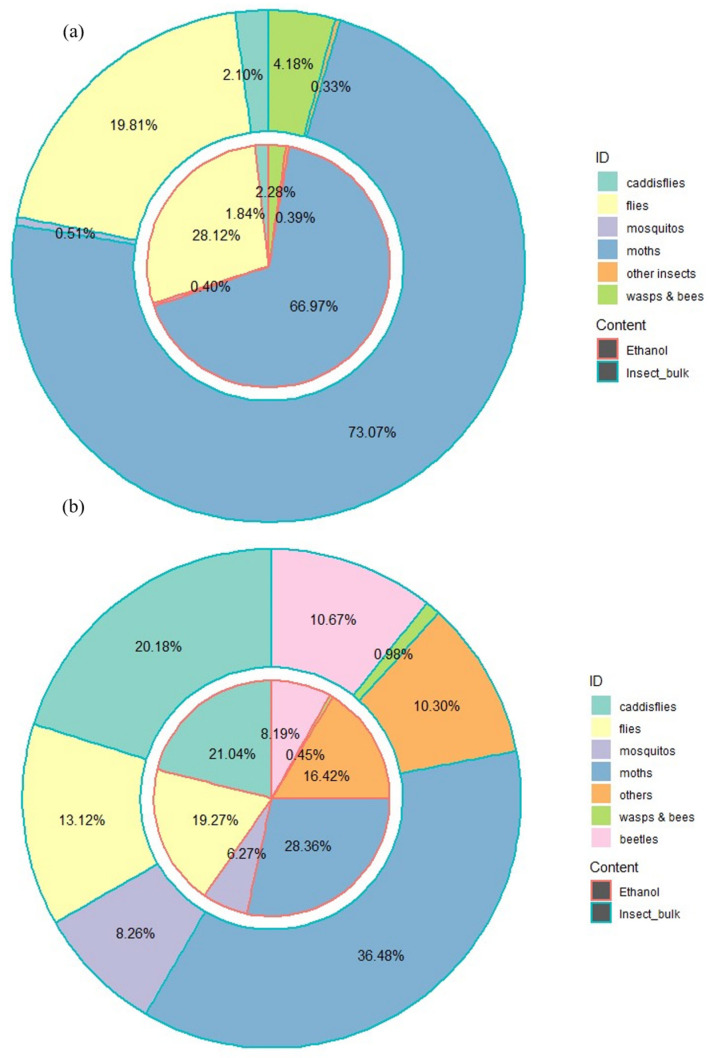
Pie charts showing the most abundant insects found in the traps identified by (**a**) the primer pair LCO1490/HCO2198 and (**b**) the primer pair mlCOIintF/jgHCO2198. The inner circle of the pie with a red outline represents eDNA extracted from trap fluid samples, while the outer circle with green outline reflects those DNA samples extracted from insect bulk. The different colours in the pie chart represent different insects. It needs to be noted that the number of sequence reads reflects the post-PCR distribution, instead of the actual species abundance or community population.

Besides species belonging to the Ceratopogonidae family, both the primer pairs LCO1490/HCO2198 and mlCOIintF/jgHCO2198 identified a variety of other insect species (Figure 5 and Figure S1). In general, the significant variation in the diversity and relative abundance of different insects recovered from the trap sample is mainly caused by the primer used, independent of the difference in the sample types (trap ethanol fluid or insect bulk). For example, a substantial proportion of the LCO1490/HCO2198 COI OTUs were taxonomically assigned to moths (i.e., order Lepidoptera); specifically, 73.07% and 66.97% of OTUs were derived from insect bulk and ethanol fluid, respectively.

However, based on the OTUs derived from mlCOIintF/jgHCO2198, that proportion drops to 36.48% and 28.36% for insect bulk and trap ethanol fluid, respectively. Moreover, flies (Diptera) were the second most abundant insect observed in both the trap ethanol fluid (28.12%) and insect bulk (19.81%) samples based on the OTUs derived from LCO1490/HCO2198. In the mlCOIintF/jgHCO2198 OTU dataset, that proportion only reached 19.27% and 13.12% for the trap ethanol fluid and insect bulk samples, respectively, making flies the third most abundant insect following the caddisflies (Trichoptera; 21.04% for ethanol fluid and 20.18% for insect bulk). More importantly, instead of one prevalent insect type acting as the most dominant order group observed in the dataset of LCO1490/HCO2198 (Figure 5a), more diverse taxonomic groups with similar proportions for each insect type were observed from the mlCOIintF/jgHCO2198 dataset (Figure 5b). Such a difference in the taxonomic diversity derived from different primer choices is more distinct at the family level (Figure S2).

### 3.5. Similar Insect Community Composition Recovered by Ethanol Fluid and Insect Bulk Samples

**Figure 6:**
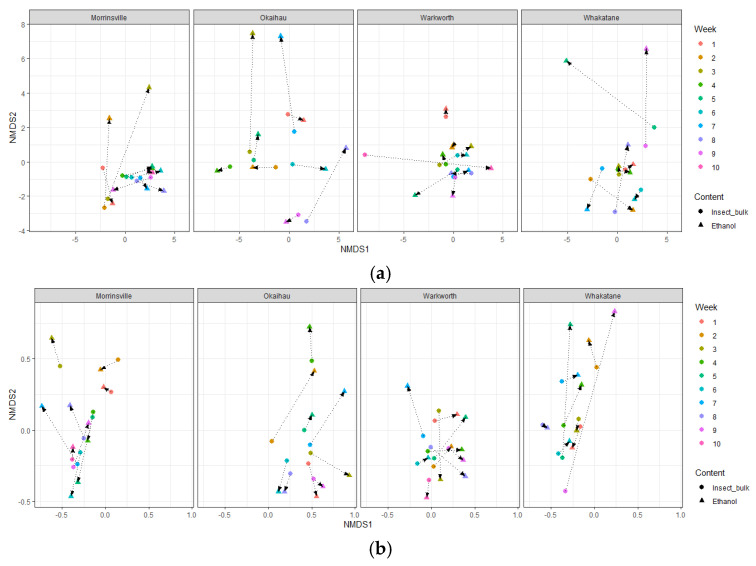
Non-metric multidimensional scaling (nMDS) plots representing variations in the composition of DNA metabarcoding data comparing samples collected from different field sites (i.e., Morrinsville, Okaihau, Warkworth, and Whakatane) at different collection dates (from week 1 to week 10) using (**a**) the primer pair LCO1490/HCO2198 and (**b**) the primer pair mlCOIintF/jgHCO2198. The nMDS plot was constructed using a Bray–Curtis dissimilarity matrix derived from COI gene data grouped into operational taxonomic groups at 97% DNA sequence similarity. Sample data closer to each other are expected to contain more similar (eDNA) insect communities. The colour of points is assigned based on the collection date. The shape of points indicates different sample types (i.e., ethanol versus insect body bulk derived from the same trap) used to extract eDNA. The samples obtained from the same site and collected at the same time are linked by lines.

In general, the insect community composition recovered from the trap samples did not significantly differ between different sample types (trap ethanol fluids versus insect bulks) or sampling sites (Figure S3), as the majority of them overlapped between the OTUs recovered from ethanol fluid and insect bulk samples. A closer look revealed that for most trap samples, their community compositions recovered from ethanol fluid and insect bulk were greatly similar when comparing the samples collected from the same site at the same collection time (Figure 6).

Furthermore, greater Bray–Curtis dissimilarity distances between the same sample derived from ethanol fluid and insect bulk were observed, based on their OTUs amplified by mlCOIintF/jgHCO2198, than for those amplified by LCO1490/HCO2198, suggesting a greater influence of primer selection on OTUs obtained from the same sample, in terms of the taxonomic diversity and composition of the whole insect community, compared with the sample types.

## 4. Discussion

In the current study, the detection accuracies of the DNA metabarcoding approaches in the surveillance of biting midges (Ceratopogonidae) were assessed using temporal samples collected over ten weeks from light traps at four different surveillance sites. This study compared DNA metabarcoding with morphological findings to determine if it could accurately detect target species and recover the taxonomic diversity of insect communities using different sample types (trap ethanol fluid versus insect bulk samples) and two COI barcoding regions for DNA metabarcoding approaches. The results indicated that sample types impacted the detection accuracy of target species of Ceratopogonidae. DNA metabarcoding using homogenized insect bulk samples correctly identified the target species observed via morphology in over 81% of trap samples, while the accuracy using eDNA from ethanol fluids was only 55–68% with two different primer sets. However, the recovery of overall taxonomic diversity and insect community composition was influenced by primer choices rather than sample types. These findings highlight opportunities to optimize diagnostics using insect bulk samples and eDNA from trap fluids for detecting unwanted insect pests and monitoring biodiversity in pest surveillance traps.

### 4.1. Detection of Target Species Is Greatly Impacted by Sample Types and Primer Selection

Using mixed homogenized insect bulk from trap samples can result in the loss of individual insects, leaving no sample for morphological examination after the extraction. While DNA extraction from homogenized insect tissues is used to study community composition [44,45,46], some other studies have suggested that eDNA extraction from preservative mediums like ethanol or saturated salt (NaCl) solution can be an alternative while preserving intact specimens for further taxonomic work [28,47,48,49].

However, metabarcoding results from preservative trap fluid may differ from those of homogenized tissues due to the disproportionate DNA contribution from larger organisms in comparison to smaller ones, subsequently biasing the proportion of HTS reads per specimen in the sample [50]. To address this, this study compares the consistency and accuracy of metabarcoding results between homogenized insect bulk and ethanol fluid samples using two COI barcoding markers, alongside morphological findings. The eDNA approach from ethanol fluid offers a non-destructive method to detect target species and investigate biodiversity.

The study of Hajibabaei et al. (2012) [47] pioneered the use of eDNA extracted from the preservative ethanol for high-throughput barcoding, achieving an ~89% recovery rate of taxa identified from insect tissue homogenate. Since then, there has been growing interest in using preservative ethanol for metabarcoding in biodiversity monitoring surveys [48,49,51,52,53]. However, its effectiveness in biosecurity surveillance, specifically targeting unwanted species, remains largely unexplored. To the best of our knowledge, this study is among the first to compare DNA metabarcoding results from both preservative ethanol and insect tissue homogenate with morphological findings in a biosecurity surveillance context with targeted species.

We found that the detection and identification of the presence of the indicator species, Ceratopogonidae species, in surveillance trap samples recovered from eDNA metabarcoding of preservative ethanol fluid was significantly less sensitive compared with those recovered from homogenized insect body bulk sample. In this case, the eDNA approach being unable to recover the DNA of target species from the ethanol fluid was the major reason causing the failure of eDNA-based detection, regardless of the relative abundance of the target species. Notably, our study is not the first to report the detection failure (e.g., false negative) of the DNA of the specific insect species from the fluid samples (e.g., water). For example, the study of Schneider et al. (2016) [31] achieved false-negative results through metabarcoding, and this was likely due to the low specimen numbers within the whole community. Similarly, Krol et al. (2019) [54] reported that only a subset of the species discovered from the trap was identified by eDNA extracted from environmental water. Similar observations were also made in other eDNA field studies targeting other organisms, such as fish [55] and other macroinvertebrates [56]. The heterogeneous distribution of eDNA in the fluid body, as well as PCR-biases, could contribute together to the detection failures that we observed in the current study.

Since the eDNA of the specific target is not evenly distributed in the fluid body [57,58], after the insect is captured and dead in the preservative ethanol in the surveillance trap, its eDNA is more likely flow around its body. In addition, *Culicoides* and Ceratopogonidae species are well known for their small body size, measuring only 1–3 mm [1]. As a consequence, they release less DNA into the ethanol, therefore representing only a small fraction of the total biomass, as well as of the eDNA in the sample [59]. Together, those impacts reduce the possibility of eDNA capture and the extraction of small insects, like Ceratopogonidae species, from the ethanol.

Furthermore, during the downstream metabarcoding amplification, such an uneven distribution of eDNA derived from the insect with different body sizes within the community could be exacerbated by PCR-biases [29,60,61], possibly leading to the underestimation or under-detection of our small-body sized target species. This could also explain why the eDNA metabarcoding conducted by the primer set mlCOIintF/jgHCO2198 produced more detection failures (i.e., false negatives) compared with those achieved using the primer set LCO1490/HCO2198.

In contrast, the detection accuracies of DNA metabarcoding approaches using the homogenized insect body bulk samples were largely consistent between the amplicon sequencing conducted by different primer sets, both of which, in general, greatly recovered the DNA of species belonging to Ceratopogonidae family from the traps. This could explain very low false negatives produced via this approach.

Besides the false negatives likely caused by the same reasons discussed above for eDNA derived from ethanol, a few false positives produced by metabarcoding could have possibly been caused by ‘cross-talk’, with the incorrect assignment of reads to the wrong samples [62]. A similar observation was previously reported by Mata et al. (2020) [63], suggesting that the DNA of some insect species, which could only be caught during late summer and early autumn, was detected from DNA extracted from the homogenized insect bulk caught by the trap placed in late spring. In an aquatic environment, a similar observation was made where bulk-sample metabarcoding identified more taxa with a higher taxonomic resolution than eDNA metabarcoding in freshwater biomonitoring [43,64,65].

While morphological identification served as the reference standard for comparing DNA metabarcoding outcomes across two sample types, it is important to acknowledge that some false positives (i.e., detection via metabarcoding but not by morphology) could be attributed to inherent limitations in the morphological approach’s ability to detect all target species. This was evident in some samples where metabarcoding detected the presence of Ceratopogonidae species in one liquid trap sample and three insect bulk samples that the morphological screening failed to identify. This discrepancy could be explained by the potential for small or fragmented body parts, possibly missed during microscopic examination, to be captured and detected through the homogenized bulk samples and trap liquid samples used for metabarcoding.

### 4.2. Consistent Insect Community Composition Data in Ethanol Versus Homogenized Samples

Although a variable number of Ceratopogonidae species catches were observed across the four surveillance sites and within samples from the same site, detection failures were minimal when the relative abundance of the target species within the community increased. This suggests a higher probability of detecting shed eDNA in the trap liquid or recovering DNA from homogenized insect bulk samples when multiple individuals of the same species are present in a trap compared to the presence of a single individual.

While targeted species detection varied between ethanol and homogenized samples, overall taxonomic diversity and insect community composition were consistent. This aligns with the results of most recent studies [48,49,66], though other studies [52,53] reported divergent estimates of community composition recovered from ethanol eDNA compared with those derived from homogenate bulk samples, likely due to their use of heavily sclerotized terrestrial insects.

Our findings, supported by Mata et al. (2020) [63], which focused on nocturnal flying insect communities using light traps (similar to our study using light traps to attract nocturnal *Culicoides* and Ceratopogonidae species), found highly similar community compositions between morphology and metabarcoding approaches derived from homogenized insect bulk samples. This suggests that insect type influences eDNA recovery from trap ethanol fluid. Further research is required to determine how trapping methods affect diversity recovery from trap fluid samples. Collectively, further experimental work is required to address whether different trapping methods, such as light traps and Malaise traps, would have influence on the diversity recovery of a non-destructive extraction protocol using the preservative ethanol from the insect trap samples.

## 5. Conclusions

In conclusion, we demonstrate that using a COI-based metabarcoding approach, homogenized insect bulk samples collected from surveillance traps can correctly detect the presence or absence of the target species identified morphologically in most trap samples (i.e., over 81%). In contrast, the preservative ethanol fluid derived from the trap provides weaker detection accuracy (i.e., 55–68%). This difference between destructive (i.e., homogenization of insect bulk samples) and non-destructive (i.e., eDNA shed from trap fluids) approaches was previously observed [52,66], likely due to variations in the amount of DNA of target species leaking into the ethanol or homogenized from insect bulk based on their biomass size and sclerotization.

Therefore, the utility of more sensitive and specific diagnosis techniques, such as quantitative PCR (qPCR), targeting only the specific unwanted species could potentially increase the detection accuracy of the non-destructive eDNA approach. However, similar insect community compositions observed from both approaches suggest that the implementation of eDNA metabarcoding using preservative ethanol fluid provides a convenient approach for the rapid and non-destructive monitoring of spatio-temporal variations in the diversity and composition of complex insect communities. This approach offers the ability to process hundreds to thousands of insect samples at high taxonomic resolution in a relatively short time frame, which is hardly achieved by conventional morphological approaches.

Overall, DNA metabarcoding using either destructive or non-destructive protocols shows the potential for improving and complementing the efficiency and early detection of invasive species in traditional surveillance. Meanwhile, this approach also demonstrates the capability to reliably recover the true insect community composition for biodiversity monitoring.

## Figures and Tables

**Table 1:** The metadata of the sampling sites and morphological outcomes from all sampling sites.

Farm Location	Week Number	Collection Date	Total Number of Insects in the Trap	Number of Native Ceratopogonidae (% of Total Insects)	If *Culicoides* Present
Okaihau	1	7 February 2020	300	0	No
	2	11 February 2020	500	0	No
	3	14 February 2020	1196	0	No
	4	24 February 2020	24,976	1	No
	5	4 March 2020	300	1	No
	6	9 March 2020	300	1	No
	7	18 March 2020	80	1	No
	8	23 March 2020	80	1	No
	9	31 March 2020	80	0	No
Total			27,812	5 (0.02%)	
Warkworth	1	7 February 2020	600	0	No
	2	12 February 2020	200	0	No
	3	19 February 2020	150	1	No
	4	25 February 2020	300	0	No
	5	28 February 2020	2598	0	No
	6	10 March 2020	300	1	No
	7	19 March 2020	1150	0	No
	8	31 March 2020	2000	2	No
	9	3 April 2020	150	0	No
	10	9 April 2020	150	0	No
Total			7598	4 (0.05%)	
Morrinsville	1	7 February 2020	1000	1	No
	2	12 February 2020	150	0	No
	3	14 February 2020	150	0	No
	4	21 February 2020	500	0	No
	5	28 February 2020	350	1	No
	6	6 March 2020	400	4	No
	7	13 March 2020	300	0	No
	8	24 March 2020	300	1	No
	9	27 March 2020	700	3	No
	10	3 April 2020	150	0	No
Total			4000	10 (0.25%)	
Whakatane	1	12 February 2020	500	8	No
	2	14 February 2020	3200	2	No
	3	24 February 2020	300	6	No
	4	28 February 2020	250	39	No
	5	6 March 2020	80	2	No
	6	13 March 2020	855	5	No
	7	23 March 2020	500	1	No
	8	27 March 2020	600	25	No
	9	9 April 2020	50	7	No
Total			6335	95 (1.5%)	

## Data Availability

All relevant data are included in this article. Raw sequencing data have been deposited in the NCBI SRA (Sequence Read Archive) database with the BioProject ID PRJNA1249277. The in-house customized COI reference database of the Ceratopogonidae species is available on request.

## Supplementary Materials

The following supporting information can be downloaded at https://www.mdpi.com/article/10.3390/insects16060564/s1, Figure S1: Pie charts showing the overall taxonomic range at the order level across the study sites; Figure S2: Pie charts showing the overall taxonomic range at the family level across the study sites; Figure S3: Non-metric multidimensional scaling (nMDS) plots showing variations in the composition of eDNA sequencing data.

## Abbreviations

The following abbreviations are used in this manuscript:

eDNA	Environmental DNA
COI	Cytochrome oxidase I
HTS	High-throughput sequencing
OTU	Operational taxonomic unit
MPI	Ministry for Primary Industries
NCBI	National Centre for Biotechnology Information
NMDS	Non-metric multidimensional scaling
PHEL	Plant Health and Environment Laboratory
SRA	Sequence Read Archive
