# Supplementary material for: Comparative Assessment of Environmental DNA and Bulk-Sample Metabarcoding in Biosecurity Surveillance for Detecting Biting Midges (Ceratopogonidae)
Source: Insects. 2025 May 27;16(6):564. doi: 10.3390/insects16060564 (PMC12193466; doi:10.3390/insects16060564)
Supplement: Supplementary file 1 [file insects-16-00564-s001.zip › insects-3622959-supplementary.pdf]

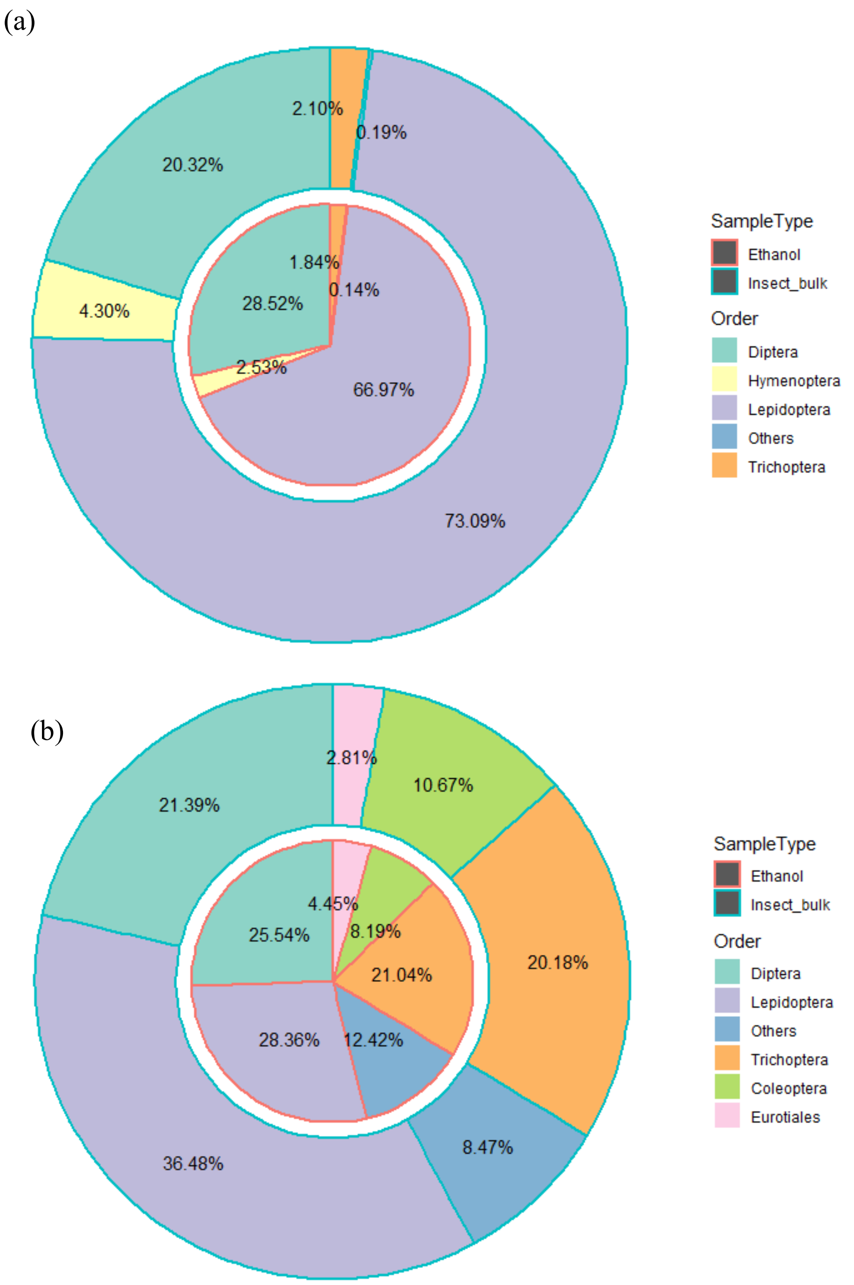

Figure S1. Pie charts showing the overall taxonomic range at order level across the study sites identified by (a) the primer pair LCO1490/HCO2198 and (b) the primer pair mlCOIintF/jgHCO2198. The inner circle of the pie and its red outline represent that those eDNA was extracted from ethanol samples, while the outer circle with green outline reflects those eDNA samples extracted from insect body bulks. The different colours in the pie chart represent different orders. (It needs to be noted that the number of sequence reads reflects the post-PCR distribution, instead of the actual species abundance or community population.)

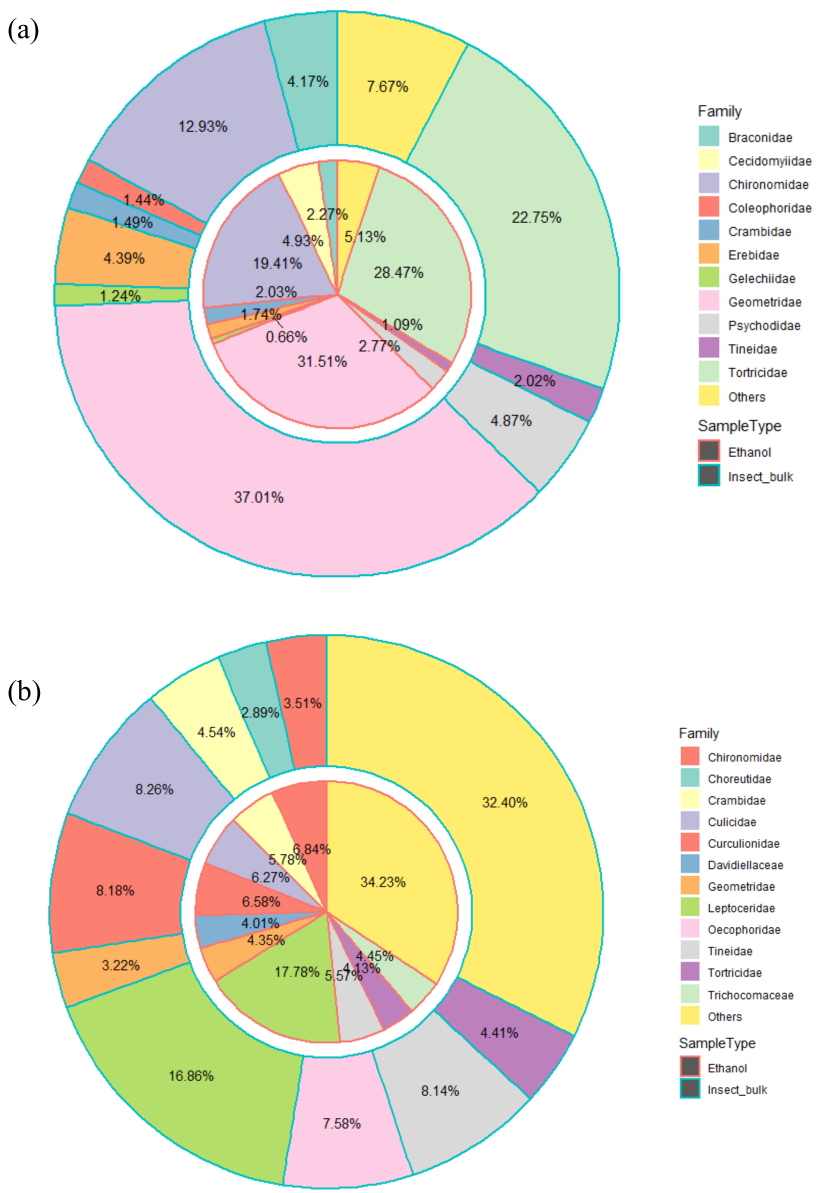

Figure S2. Pie charts showing the overall taxonomic range at family level across the study sites identified by (a) the primer pair LCO1490/HCO2198 and (b) the primer pair mlCOIintF/jgHCO2198. The inner circle of the pie and its red outline represent that those eDNA was extracted from ethanol samples, while the outer circle with green outline reflects those eDNA samples extracted from insect body bulks. The different colours in the pie chart represent different families. (It needs to be noted that the number of sequence reads reflects the post-PCR distribution, instead of the actual species abundance or community population.)

(a)

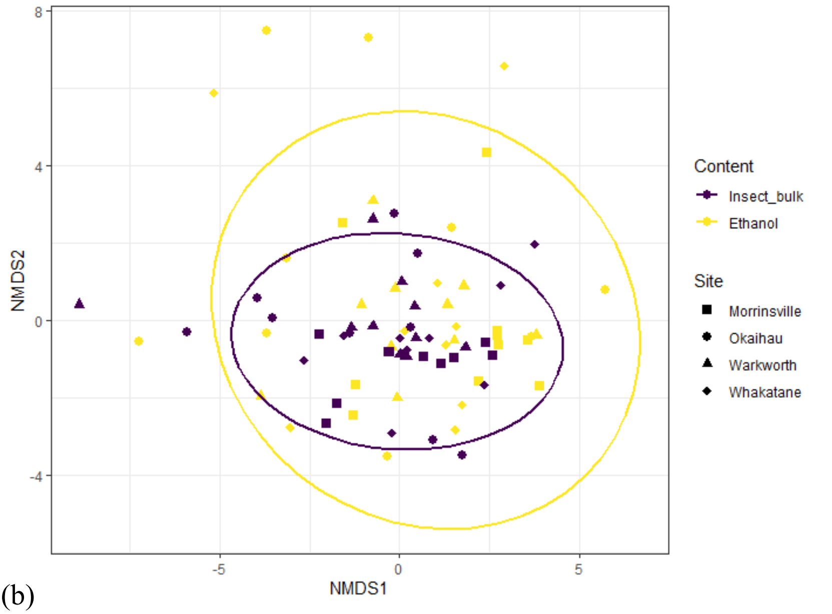

(b)

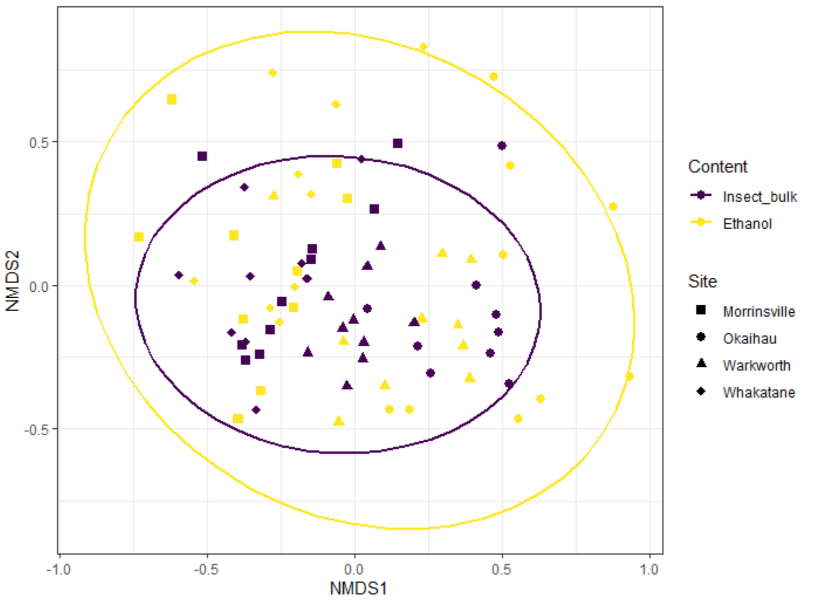

Figure S3. Non-metric multidimensional scaling (nMDS) plots showing variations in the composition of eDNA sequencing data comparing samples collected from different field sites (i.e., Morrinsville, Okaihau, Warkworth and Whakatane) based on different sample types (i.e., ethanol versus insect body bulk derived from the same trap) using (a) the primer pair LCO1490/HCO2198 and (b) the primer pair mlCOIintF/jgHCO2198. The nMDS plot was constructed a using Bray-Curtis dissimilarity matrix derived from COI gene data grouped into operational taxonomic groups at 97% DNA sequence similarity. Sample data closer to each other are expected to contain more similar (eDNA) insect communities. The colour of points are assigned based on the sample types (i.e., ethanol or insect bulk). The shape of points indicate different sampling site (i.e., ethanol versus insect body bulk) used to extract eDNA. The ellipses show the assumed multivariate t-distribution at the centre of each group of each land type at a 95% confidence level.
